# Artificial Neural Network Accurately Predicts Hepatitis B Surface Antigen Seroclearance

**DOI:** 10.1371/journal.pone.0099422

**Published:** 2014-06-10

**Authors:** Ming-Hua Zheng, Wai-Kay Seto, Ke-Qing Shi, Danny Ka-Ho Wong, James Fung, Ivan Fan-Ngai Hung, Daniel Yee-Tak Fong, John Chi-Hang Yuen, Teresa Tong, Ching-Lung Lai, Man-Fung Yuen

**Affiliations:** 1 Department of Infection and Liver Diseases, Liver Research Center, the First Affiliated Hospital of Wenzhou Medical University, Wenzhou, Zhejiang, China; 2 Department of Medicine, the University of Hong Kong, Queen Mary Hospital, Hong Kong, China; 3 Department of Nursing Studies, the University of Hong Kong, Queen Mary Hospital, Hong Kong, China; 4 State Key Laboratory for Liver Research, the University of Hong Kong, Queen Mary Hospital, Hong Kong, China; CRCL-INSERM, France

## Abstract

**Background & Aims:**

Hepatitis B surface antigen (HBsAg) seroclearance and seroconversion are regarded as favorable outcomes of chronic hepatitis B (CHB). This study aimed to develop artificial neural networks (ANNs) that could accurately predict HBsAg seroclearance or seroconversion on the basis of available serum variables.

**Methods:**

Data from 203 untreated, HBeAg-negative CHB patients with spontaneous HBsAg seroclearance (63 with HBsAg seroconversion), and 203 age- and sex-matched HBeAg-negative controls were analyzed. ANNs and logistic regression models (LRMs) were built and tested according to HBsAg seroclearance and seroconversion. Predictive accuracy was assessed with area under the receiver operating characteristic curve (AUROC).

**Results:**

Serum quantitative HBsAg (qHBsAg) and HBV DNA levels, qHBsAg and HBV DNA reduction were related to HBsAg seroclearance (P<0.001) and were used for ANN/LRM-HBsAg seroclearance building, whereas, qHBsAg reduction was not associated with ANN-HBsAg seroconversion (P = 0.197) and LRM-HBsAg seroconversion was solely based on qHBsAg (P = 0.01). For HBsAg seroclearance, AUROCs of ANN were 0.96, 0.93 and 0.95 for the training, testing and genotype B subgroups respectively. They were significantly higher than those of LRM, qHBsAg and HBV DNA (all P<0.05). Although the performance of ANN-HBsAg seroconversion (AUROC 0.757) was inferior to that for HBsAg seroclearance, it tended to be better than those of LRM, qHBsAg and HBV DNA.

**Conclusions:**

ANN identifies spontaneous HBsAg seroclearance in HBeAg-negative CHB patients with better accuracy, on the basis of easily available serum data. More useful predictors for HBsAg seroconversion are still needed to be explored in the future.

## Introduction

In clinical practice, hepatitis B surface antigen (HBsAg) seroclearance and seroconversion have been recommended as the ideal outcomes in both the natural history of HBV infection and as endpoint for the treatment of CHB [1]. Earlier HBsAg seroclearance or seroconversion is likely resulted in a better prognosis because of lower HBV replication as well as less liver damage [1], [2]. A few studies have explored the incidence of spontaneous HBsAg seroclearance in CHB patients of both Asian and European populations using long-term follow-up cohorts and the annual incidence ranges from 0.62% to 2.26% [3], [4], [5], [6], [7], [8]. Because of the more rarity of spontaneous HBsAg seroconversion, compared to HBsAg seroclearance, the incidence and long-term outcomes of CHB patients experiencing this event remain disputed. Existing evidences indicate that HBsAg seroclearance or seroconversion confers favorable long-term outcomes in patients without hepatocellular carcinoma (HCC) or decompensated liver cirrhosis [9], [10], [11], [12].

Predictive factors for spontaneous HBsAg seroclearance or seroconversion using various parameters have attracted much attention recently. Previous studies had demonstrated that lowering HBV DNA level was an important predictor for spontaneous HBsAg seroclearance [5], [6], [8], [13]. Furthermore, with the technological advances of quantitative HBsAg (qHBsAg), it has been suggested as a promising new marker in monitoring immunological response in both treated and untreated CHB patients, as well as a potential predictor of liver disease progression [14]. Our previous study showed that low qHBsAg levels and an increased reduction rate in qHBsAg levels were the most significant predictors of spontaneous HBsAg seroclearance with 3 years of follow-up [15]. These findings have been further validated by other studies [4], [5], [13], [16], [17]. However, our previous study had several limitations. No specific time point was identified where qHBsAg kinetics could have the highest predictive value. Also the accuracy of qHBsAg levels in predicting HBsAg seroclearance [area under receiver operating characteristic curve (AUROC) 0.833] still warrants improvement [15]. In all currently available studies [4], [5], [13], [15], [16], [17], the predictability of qHBsAg levels for HBsAg seroconversion has not been thoroughly investigated.

Being a complex biological system, the interactions among predictors are multidimensional and non-linear, thus, making it difficult to distinguish between classes when using the conventional linear discriminant analysis or a single predictor. The artificial neural network (ANN) is a novel computer model inspired by the working of the human brain [18]. It consists of a set of highly interconnected processing units (neurons) linked with weighted connections, and includes an input layer, an output layer and one or more hidden layers. The input layer is formed from the different data available for the analysis and the output layer is formed from the different outcomes, whereas, the hidden layers are used to allow complex relations between the input and output layers to evolve. One of the outstanding characteristics of the ANN is that it can develop nonlinear statistical models to deal with complex biological systems [19].

The main aim of the present study was to assess the ability of the ANNs to predict HBsAg seroclearance and seroconversion in a large population of CHB patients spontaneously clearing HBsAg with or without the appearance of anti-HBs and compared ANNs performance to that of conventional logistic regression models (LRMs) as well as previously proven clinical parameters, such as qHBsAg and HBV DNA levels.

## Materials and Methods

The composition of the present study cohort has been previously described, and is based on the comparison of CHB patients with spontaneous HBsAg seroclearance, with age- and sex-matched HBeAg-negative controls [15]. The present study was a post-hoc analysis involving the entire cohort of our previous study. In brief, all of the patients were followed up at the Liver Clinic, Department of Medicine, the University of Hong Kong, Queen Mary Hospital regularly for at least 3 years. All patients had HBsAg positivity documented for more than six months and were HBeAg-negative on presentation to our clinic. Upon their first and/or follow-up visits, these patients had given verbal informed consent for the storage of blood samples for further studies.

HBsAg seroclearance or seroconversion was observed in the first group of patients between June 2001 and February 2011; these patients were then followed up regularly until June 2012 for their latest liver biochemistry and HBV serology. HBsAg seroclearance was defined as loss of serum HBsAg with or without the appearance of antibody to HBsAg (anti-HBs), while HBsAg seroconversion was defined as loss of serum HBsAg with the appearance of anti-HBs. These two end-points were confirmed by two samples taken at least six months apart. The control group, recruited between May 2010 and May 2011, was age- and sex-matched with the patient group achieving HBsAg seroclearance. No treatment had been given for all of the patients during the entire follow-up period. Serum samples collected at every visit were stored at −20°C until tested. Serum HBV DNA and qHBsAg levels were performed 3 years, 2 years before HBsAg seroclearance and at time of HBsAg seroclearance (i.e., baseline). The numbers of stored serum available for HBsAg seroclearance or seroconversion group were 203, 190 and 203 at the time points of 3 years, 2 years before and at the time of HBsAg seroclearance respectively. The corresponding numbers of stored serum available for the control groups were 203, 189 and 197.

Serum qHBsAg level was measured by the Elecsys HBsAg II assay (Roche Diagnostics, Gmbh, Mannheim, Germany) [20], with a lower limit of detection of 0.05 IU/mL. Samples with qHBsAg level higher than 52000 IU/mL were retested at a dilution of 1∶100, according to the manufacturer's instructions. Serum anti-HBs were measured by Abbott Laboratories (Chicago, Illinois), with a lower limit of detection of 10 mIU/mL. Serum HBV DNA level was measured using the Cobas Taqman assay (Roche Diagnostics, Branchburg, New Jersey), with a lower limit of detection of 20 IU/mL.

One hundred randomly chosen patients with HBsAg seroclearance, followed by 100 age- and sex-matched controls, were chosen for the determination of HBV genotype using the INNOLIPA HBV genotyping assay, which was performed according to the instructions of the manufacturer (Innogenetics, Gent, Belgium).

### Ethics Statement

Verbal informed consent was obtained and recorded among all patients upon their first and/or subsequent follow-up visits for the storage of blood samples for further studies. The study was approved by the Institutional Review Board, the University of Hong Kong and West Cluster of Hospital Authority, Hong Kong, including for the retrieval of archived samples for the present study. All clinical investigation was conducted according to the principles expressed by the Declaration of Helsinki, with all data anonymously analyzed.

### Statistical Analysis

Categorical variables were reported as the number of cases and percentages; continuous variables were explored for parametric distribution using the Kolmogorov-Smirnov test. For patients with undetectable serum HBV DNA or qHBsAg, the results were taken as the lower limit of detection (20 and 0.05 IU/mL, respectively). As HBV DNA and qHBsAg levels showed a highly skewed distribution, they were log transformed (log10) before the analysis. After transformation, both variables showed a normal distribution (P>0.05). Differences in clinical and laboratory data, related to HBsAg seroclearance or seroconversion, were assessed using the chi-square analysis with Yates correction and the independent-sample T-test procedure after Levene's test for equality of variances, as appropriate. A subgroup analysis according to different genotype of HBV was also performed to further test the power of established models.

### Development of the artificial neural network

Variables found to be significantly related to HBsAg seroclearance or seroconversion by univariate analyses were used to build the ANNs. Patients were randomly assigned to a training group (70% of total patients) or a testing group (30% of total patients). We built a three layer feed forward neural network with two output neurons. The learning rule used here was back propagation of errors, which adjusts the internal parameters of the network over the repeated training cycles to reduce the overall error [21]. The weight of the connections was also altered between neurons to decrease the overall errors of the network. Training was terminated when the sum of square errors was at a minimum. The activation function, representing the outcomes of ANN, was used with continuous outputs with the interval from 0 to 1, in which 0 = HBsAg non-seroclearance/non-seroconversion, 1 = HBsAg seroclearance/seroconversion. The cut-offs of ANN outputs with the best relationship between sensitivity and specificity were used for classification. The relative weights of the input variables for the ANNs were calculated according to the General Influence Measure method [22]. In this study, we built ANNs by using the graphical neural network development tool NeuroSolution V5.05 (Neurodimension, Gainesville, FL, USA).

### Development of the multivariate logistic regression model

In the training group (70% of total patients), variables found to be significantly related to HBsAg seroclearance or seroconversion by univariate analysis entered into two distinct forward conditional multivariate logistic regression models (LRMs). Logistic regression generated the coefficients of a formula to predict a logit transformation of the probability of presence of the characteristic of interest: logit(p)  =  b_0_ + b_1_x_1_ + b_2_x_2_ + … + b_k_x_k_. The probability of presence of the characteristic of interest was obtained by the formula *p* = 1/(1+e^−logit(p)^) in which 0 = HBsAg non-seroclearance/non-seroconversion, 1 = HBsAg seroclearance/seroconversion. The cut-offs of logistic regression outputs with the best relationship between sensitivity and specificity were adopted for classification.

### Assessment of the diagnostic accuracy

The performances of both ANNs and LRMs, as well as qHBsAg and HBV DNA levels, in predicting HBsAg seroclearance or seroconversion in the training group and in three validation groups (testing group, genotype B group, genotype C group) were tested using receiver operating characteristic (ROC) curve analysis and expressed in terms of sensitivity, specificity, positive predictive values (PPV) and likelihood ratios (LR). Youden index was calculated to discriminate the optimal cut-off value. Comparison of ROC curves was obtained using the Hanley-McNeil method [23].

A two-sided P value of<0.05 was considered statistically significant. Statistical analysis and ROC analysis were computed by MedCalc 10.0 software (Mariakerke, Belgium) and SPSS 18.0 software (SPSS Inc, Chicago, IL, USA).

## Results

### Baseline Characteristic of Patients

Baseline characteristic of the entire study population were outlined in Table 1. The mean age was 48.8±10.9 years and patients were predominantly male (70.4%). 63 patients (31.0%) in the HBsAg seroclearance group had developed anti-HBs. There were no significant differences in the distribution of age, gender, alanine aminotransferase (ALT) level, bilirubin and genotype when comparing patients with HBsAg seroclearance with and without seroconversion (all P>0.05). Patients with HBsAg seroclearance or seroconversion had significantly lower serum qHBsAg, HBV DNA levels at baseline (all P<0.001), compared to controls as previously described.[15] Specific characteristics of four groups/subgroups (including training, testing, genotype B and genotype C) were outlined in Table 2, Table S1–S5. There were no significant differences in the distribution of age, gender, ALT, bilirubin, qHBsAg level, HBV DNA, qHBsAg reduction, HBV DNA reduction between the training group with the other three testing groups (all P>0.05).

**Table 1:** Baseline characteristics of the study population.

Variables	All patients (n = 406)	HBsAg seroclearance patients (n = 203)	Control cohort (n = 203)	P
Time point 0 year (baseline)				
Age (years)	48.8±10.9	48.7±11.1	49.0±10.7	0.791
Male gender (%)	286 (70.4)	143 (70.4)	143 (70.4)	0.999
ALT (IU/L)	27.5±16.8	28.6±19.8	26.5±13.0	0.208
Bilirubin (µmol/L)	13.5±8.8	13.7±10.8	13.3±6.2	0.706
Genotype§ (%)				
B	141 (72.7)	59 (64.8)	82 (79.6)	0.244
C	53 (27.3)	32 (35.2)	21 (20.4)	
qHBsAg (log10 IU/ml)	0.52±2.02	−1.30±0.00	2.35±1.20	0.001
HBV DNA (log10 IU/ml)	2.38±1.46	1.37±0.23	3.40±1.47	0.001
Time point 2 years				
qHBsAg (log10 IU/ml)	1.59±1.45	0.56±0.93	2.60±1.12	0.001
HBV DNA (log10 IU/ml)	2.63±1.35	1.83±0.75	3.42±1.35	0.001
Time point 3 to 2 years				
qHBsAg reduction (log10 IU/ml)	0.41±0.55	0.66±0.60	0.16±0.36	0.001
HBV DNA reduction (log10 IU/ml)	0.18±0.97	0.40±0.86	−0.05±1.01	0.001
Time point 3 years				
qHBsAg (log10 IU/ml)	1.99±1.33	1.26±1.11	2.72±1.10	0.001
HBV DNA (log10 IU/ml)	2.79±1.28	2.25±0.97	3.33±1.32	0.001

§ Tested in 200 patients (194 had amplificable polymerase chain reaction products). Time point is defined as the period before HBsAg seroclearance: 0 year indicates date of seroclearance (baseline).

**Table 2:** Characteristics of the study population stratified by ANN groups.

Variables		Training group	Testing group	P
Age (years)	HBsAg seroclearance	48.4±10.8	49.7±11.0	0.288
	HBsAg seroconversion	48.3±10.5	49.4±12.3	0.536
Male gender (%)	HBsAg seroclearance	200 (70.4)	86 (70.5)	0.989
	HBsAg seroconversion	97 (70.8)	46 (69.7)	0.871
ALT (IU/L)	HBsAg seroclearance	28.0±17.4	26.3±15.2	0.356
	HBsAg seroconversion	29.3±19.2	27.0±21.2	0.445
Bilirubin (µmol/L)	HBsAg seroclearance	13.6±9.2	13.2±7.7	0.674
	HBsAg seroconversion	12.6±8.7	16.0±13.9	0.072
qHBsAg (log10 IU/ml)*	HBsAg seroclearance	1.94±1.33	2.13±1.31	0.190
	HBsAg seroconversion	1.22±1.13	1.35±1.09	0.452
HBV DNA (log10 IU/ml)*	HBsAg seroclearance	2.74±1.21	2.91±1.41	0.241
	HBsAg seroconversion	2.23±0.91	2.30±1.10	0.671
qHBsAg (log10 IU/ml)§	HBsAg seroclearance	1.53±1.43	1.72±1.50	0.250
	HBsAg seroconversion	0.57±0.96	0.54±0.88	0.847
HBV DNA (log10 IU/ml)§	HBsAg seroclearance	2.53±1.28	2.86±1.49	0.028
	HBsAg seroconversion	1.79±0.71	1.93±0.83	0.247
qHBsAg reduction (log10 IU/ml)¶	HBsAg seroclearance	0.40±0.52	0.42±0.62	0.816
	HBsAg seroconversion	0.62±0.59	0.74±0.60	0.218
HBV DNA reduction (log10 IU/ml)¶	HBsAg seroclearance	0.22±0.94	0.08±1.03	0.219
	HBsAg seroconversion	0.43±0.84	0.35±0.90	0.542

The number in training group was 284 patients related to HBsAg seroclearance and 137 patients related to HBsAg seroconversion, while in testing group was 122 patients related to HBsAg seroclearance and 66 patients related to HBsAg seroconversion. *Time point 3 years. ^§^Time point 2 years. ^¶^Time point 3 to 2 years. Time point is defined as the period before HBsAg seroclearance: 0 year indicates date of seroclearance (baseline).

### Development of the ANNs and LRMs

**Figure 1:**
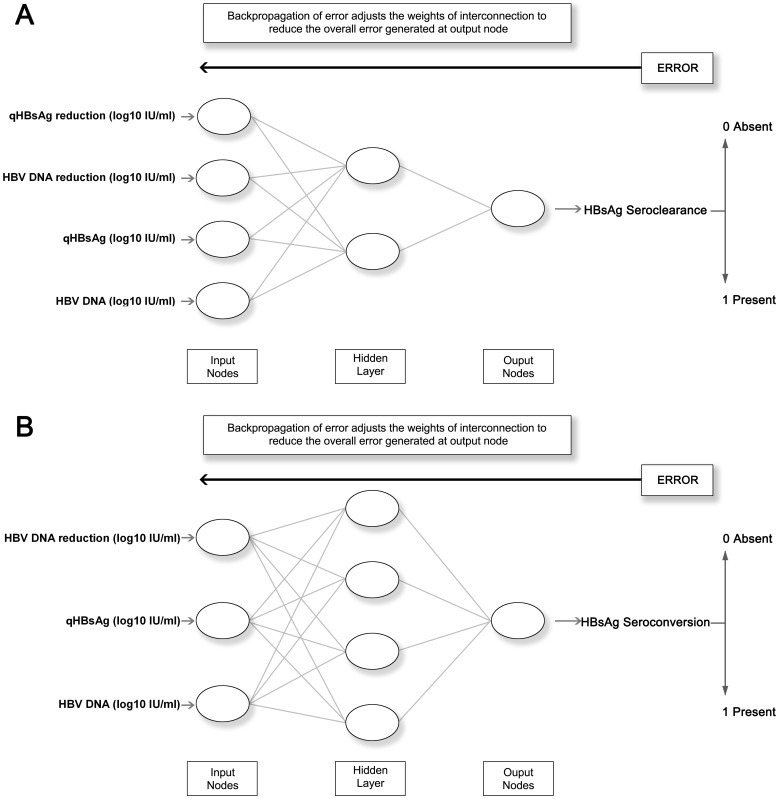
Schematic representation of the artificial neural network developed to predict A) HBsAg seroclearance and B) HBsAg seroconversion. Note: qHBsAg, 3 years before baseline; HBV DNA, 3 years before baseline; qHBsAg reduction, 3 to 2 years before baseline; HBV DNA reduction, 3 to 2 years before baseline.

**Figure 2:**
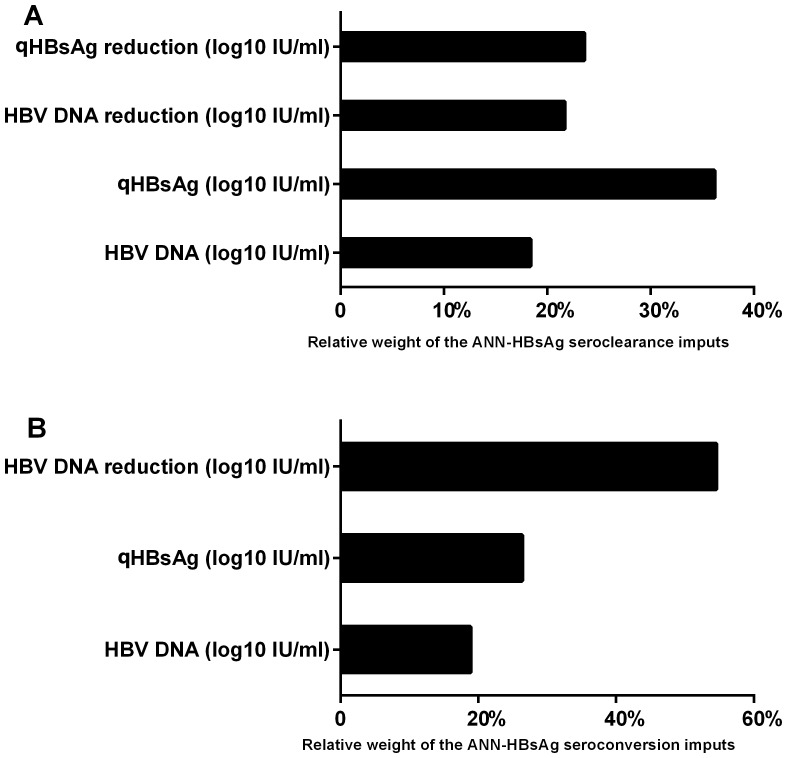
Relative weights of the clinical input parameters in an artificial neural network (ANN) trained with patients of training set. (A) ANN for HBsAg seroclearance; (B) ANN for HBsAg seroconversion. Note: qHBsAg, 3 years before baseline; HBV DNA, 3 years before baseline; qHBsAg reduction, 3 to 2 years before baseline; HBV DNA reduction, 3 to 2 years before baseline.

With respect to HBsAg seroclearance, in the training group of 284 (70% × 406) patients, qHBsAg level (OR = 0.327, 95%CI = 0.261–0.411, P<0.001), HBV DNA (OR = 0.449, 95%CI = 0.367–0.548, P<0.001), qHBsAg reduction (OR = 12.763, 95%CI = 6.575–24.773, P<0.001), and HBV DNA reduction (OR = 1.738, 95%CI = 1.356–2.227, P<0.001) were significantly associated with HBsAg seroclearance by univariate analysis (Table 3). With respect to HBsAg seroconversion, in the training group of 137 (70% × 203) patients, qHBsAg level (OR = 1.474, 95%CI = 1.112–1.953, P = 0.007), HBV DNA (OR = 1.416, 95%CI = 1.045–1.919, P = 0.025), and HBV DNA reduction (OR = 1.459, 95%CI = 1.011–2.107, P = 0.044) were significantly associated with HBsAg seroconversion by univariate analysis (Table 3). These variables were used to build the ANNs, respectively (Figure 1A and 1B). All variables had a significant contribution in predicting HBsAg seroclearance or seroconversion and the removal of any one of them diminished the performance of the ANNs (Figure 2).

**Table 3:** Clinical demographics of the training group of 284 patients related to HBsAg seroclearance and 137 patients related to HBsAg seroconversion.

Variables		Univariate analysis				Multivariate analysis			
		b	OR	95% CI	P	b	OR	95% CI	P
Age (years)	HBsAg seroclearance	−0.002	0.998	0.980–1.016	0.790	-	-	-	-
	HBsAg seroconversion	−0.030	0.971	0.937–1.005	0.096	-	-	-	-
Male gender (%)	HBsAg seroclearance	−0.091	0.913	0.547–1.523	0.727	-	-	-	-
	HBsAg seroconversion	0.069	1.071	0.556–2.063	0.837	-	-	-	-
ALT (IU/L)	HBsAg seroclearance	0.008	1.008	0.996–1.019	0.209	-	-	-	-
	HBsAg seroconversion	0.010	1.010	0.995–1.025	0.187	-	-	-	-
Bilirubin (µmol/L)	HBsAg seroclearance	0.004	1.004	0.982–1.027	0.705	-	-	-	-
	HBsAg seroconversion	−0.014	0.986	0.954–1.019	0.408	-	-	-	-
qHBsAg (log10 IU/ml)*	HBsAg seroclearance	−1.118	0.327	0.261–0.411	0.001	−1.154	0.316	0.227–0.439	0.001
	HBsAg seroconversion	0.388	1.474	1.112–1.953	0.007	0.386	1.472	1.097–1.975	0.010
HBV DNA (log10 IU/ml)*	HBsAg seroclearance	−0.802	0.449	0.367–0.548	0.001	−0.749	0.473	0.325–0.687	0.001
	HBsAg seroconversion	0.348	1.416	1.045–1.919	0.025	-	-	-	-
qHBsAg reduction (log10 IU/ml)¶	HBsAg seroclearance	2.547	12.763	6.575–24.773	0.001	2.934	18.802	7.919–44.641	0.001
	HBsAg seroconversion	0.347	1.415	0.835–2.398	0.197	-	-	-	-
HBV DNA reduction (log10 IU/ml)¶	HBsAg seroclearance	0.553	1.738	1.356–2.227	0.001	0.836	2.306	1.453–3.662	0.001
	HBsAg seroconversion	0.378	1.459	1.011–2.107	0.044	-	-	-	-

Only variables, significantly related to HBsAg seroclearance and seroconversion in the univariate analysis, were used to build the neural network, entering in the multivariate analysis for the development of the logistic regression models. Constant coefficients of multivariate logistic regression for HBsAg seroclearance  = 2.910, for HBsAg seroconversion  = −1.371.

*Time point 3 years.

¶ Time point 3 to 2 years. b, regression coefficients. CI, confidence interval.

The multivariate LRM confirmed qHBsAg level (OR = 0.316, 95%CI = 0.227–0.439, P<0.001), HBV DNA (OR = 0.473, 95%CI = 0.325–0.687, P<0.001), qHBsAg reduction (OR = 18.802, 95%CI = 7.919–44.641, P<0.001), and HBV DNA reduction (OR = 2.306, 95%CI = 1.453–3.662, P<0.001) as independent predictors for HBsAg seroclearance (284 patients); qHBsAg level (OR = 1.472, 95%CI = 1.097–1.975, P = 0.01) as independent predictors for HBsAg seroconversion (137 patients), and were used to build the LRMs, respectively (Table 3).

### Assessment of the predictive accuracy of ANNs compared with LRMs/parameters

**Figure 3:**
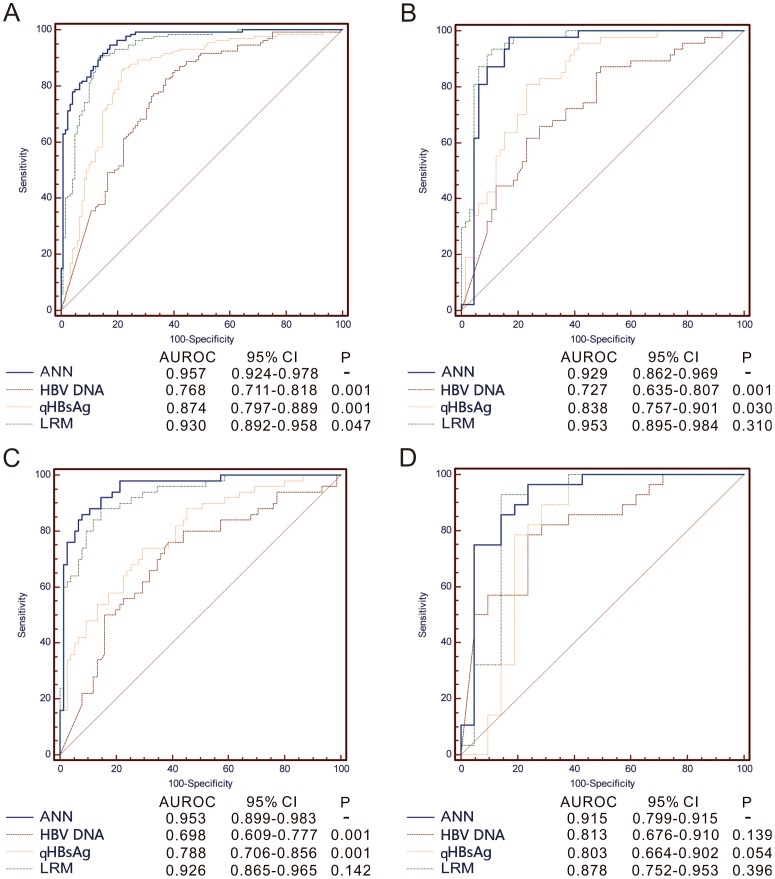
ROC analysis displaying the ability of four models/parameters (ANN, LRM, qHBsAg* and HBV DNA*) to discriminate HBsAg seroclearance in (A) training group; (B) testing group; (C) genotype B subgroup; (D) genotype C subgroup. *Time point 3 years; LRM, logistic regression model; ANN, artificial neural network.

The performance of the ANN in predicting HBsAg seroclearance in this group was very high, with AUROC of 0.957 (95%CI = 0.924–0.978). It was significantly higher compared to that of the LRM, qHBsAg level and HBV DNA (AUROC 0.930, 95%CI = 0.892–0.958, P = 0.047; 0.847, 95%CI = 0.797–0.889, P<0.001; 0.768, 95%CI = 0.711–0.818, P<0.001 respectively) (Figure 3A, Table 4). Although the AUROC of the ANN in predicting HBsAg seroconversion was lower than that for HBsAg seroclearance, the performance of the ANN in predicting HBsAg seroconversion was still better than the LRM, qHBsAg level and HBV DNA. It was 0.757 (95%CI = 0.672–0.829) which was still significantly higher than that of HBV DNA (AUROC 0.604, 95%CI = 0.513–0.690; P = 0.013), and showed a trend to be better than those of LRM and qHBsAg level (AUROC 0.670, 95%CI = 0.581–0.751, P = 0.063; 0.670, 95%CI = 0.581–0.751, P = 0.063 respectively) (Table 4). With a cut-off value of 0.474, ANN-HBsAg seroclearance had an excellent sensitivity of 94.7% and specificity of 82.6% (Table 5, data of ANN-HBsAg seroconversion not shown).

**Table 4:** Receiver-operating characteristic analysis of the artificial neural network in comparison with logistic regression model, as well as qHBsAg and HBV DNA.

		ANN		LRM		qHBsAg (log10 IU/ml)		HBV DNA (log10 IU/ml)	
		AUROC	95% CI	AUROC	95% CI	AUROC	95% CI	AUROC	95% CI
Training group	HBsAg seroclearance¶	0.957	0.924–0.978	0.930	0.892–0.958	0.847	0.797–0.889	0.768	0.711–0.818
	HBsAg seroconversion†	0.757	0.672–0.829	0.670	0.581–0.751	0.670	0.581–0.751	0.604	0.513–0.690
Testing group	HBsAg seroclearance*	0.929	0.862–0.969	0.953	0.895–0.984	0.838	0.757–0.901	0.727	0.635–0.807
	HBsAg seroconversion‡	0.626	0.494–0.746	0.556	0.424–0.682	0.556	0.424–0.682	0.549	0.418–0.676
Genotype B group	HBsAg seroclearance£	0.953	0.899–0.983	0.926	0.865–0.965	0.788	0.706–0.856	0.698	0.609–0.777
	HBsAg seroconversion§	0.784	0.649–0.885	0.649	0.505–0.775	0.649	0.505–0.775	0.611	0.467–0.742
Genotype C group	HBsAg seroclearance#	0.915	0.799–0.975	0.878	0.752–0.953	0.803	0.664–0.902	0.813	0.676–0.910
	HBsAg seroconversion¥	0.529	0.336–0.716	0.549	0.355–0.733	0.549	0.355–0.733	0.603	0.405–0.778

ANN, artificial neural network. LRM, logistic regression model. AUROC, area under the receiver operating characteristic curve. CI, confidence interval. qHBsAg, 3 years before baseline; HBV DNA, 3 years before baseline. Number of patients analyzed: ¶ 284; † 137; * 122; ‡ 66; £ 141; § 59; # 53; ¥ 32.

**Table 5:** Sensitivity, specificity, predictive values and likelihood ratios of models according to optimal cut-off for predicting HBsAg seroclearance.

	Optimal cut-off	Sensitivity	Specificity	PPV	NPV	LR+	LR−
ANN	0.474	94.7%	82.6%	85.6%	93.5%	5.46	0.064
LRM	0.471	90.2%	86.8%	88.1%	89.0%	6.82	0.11
qHBsAg (log10 IU/ml)	0.449	83.4%	75.2%	79.2%	80.0%	3.36	0.22
HBV DNA (log10 IU/ml)	0.423	82.8%	60.2%	70.2%	75.5%	2.08	0.29

PPV, positive predictive value. NPV, negative predictive value. LR+, positive likelihood ratio. LR−, negative likelihood ratio. ANN, artificial neural network. LRM, logistic regression model. qHBsAg, 3 years before baseline; HBV DNA, 3 years before baseline.

### Validation in testing group

When the ANNs were evaluated in the testing group, the performance of the ANN in predicting HBsAg seroclearance was very high, with AUROC of 0.929 (95%CI = 0.862–0.969) which was significantly higher than qHBsAg level and HBV DNA (AUROC 0.838, 95%CI = 0.757–0.901, P = 0.030; 0.727, 95%CI = 0.635–0.807, P<0.001). It was comparable to that of the LRM (AUROC 0.953, 95%CI = 0.895–0.984; P = 0.310) (Figure 3B, Table 4). The performance of the ANN in predicting HBsAg seroconversion was not satisfactory, with an AUROC of 0.626 (95%CI = 0.494–0.746). It was not significantly better when compared to that of the LRM, qHBsAg level and HBV DNA (AUROC 0.556, 95%CI = 0.424–0.682, P = 0.308; 0.556, 95%CI = 0.424–0.682, P = 0.308; 0.549, 95%CI = 0.418–0.676, P = 0.311) (Table 4).

### Validation in the subgroup of genotype B and C

The performance of the ANN in predicting HBsAg seroclearance in genotype B subgroup (141 patients) was very high, with AUROC of 0.953 (95%CI = 0.899–0.983) significantly higher than qHBsAg level and HBV DNA (AUROC 0.788, 95%CI = 0.706–0.856, P<0.001; 0.698, 95%CI = 0.609–0.777, P<0.001). It was comparable to that of the LRM (AUROC 0.926, 95%CI = 0.865–0.965; P = 0.142) (Figure 3C, Table 4). In genotype C subgroup (53 patients), the performance of the ANN in predicting HBsAg seroclearance was higher than LRM, qHBsAg level and HBV DNA. However, it did not draw a statistically significance among them (P = 0.396, P = 0.054, P = 0.139, respectively) (Figure 3D, Table 4). Similarity, the performance of the ANN in predicting HBsAg seroconversion in genotype B and C subgroups (59 and 32 patients, respectively) was not satisfactory, with an AUROC of 0.784 (95%CI = 0.649–0.885) and 0.529 (95%CI = 0.336–0.716), respectively, not significantly higher than that of the LRM, qHBsAg level and HBV DNA (P = 0.055, P = 0.055, P = 0.053, respectively; P = 0.846, P = 0.846, P = 0.441, respectively) (Table 4).

## Discussion

HBsAg seroclearance and seroconversion are accepted worldwide as the two most powerful indictors of prognosis in CHB patients, as shown by many studies investigating these topics [2], [9], [10], [11], [12], [24]. Prejudging or predicting of these features to untreated or treated CHB patients are therefore, highly desirable, as they could help hepatologists in providing optimal therapeutic regimen [1].

In recent years, ANN modeling has been increasingly used in clinical management and disease prognostication, including in the prediction of disease-free survival in HCC patients [25], assessing preoperative HCC grading and micro-vascular invasion [26], and predicting the mortality risk of patients with end-stage liver disease or acute-on-chronic hepatitis B liver failure [27], [28]. Due to the three main advantages of ANN, namely self-learning, self-adapting and inference process, the ANN model has been demonstrated to perform better than conventional discriminant analysis in precisely predicting disease outcomes [19]. To date, the complex interaction of the different variables that can be obtained during the natural history of CHB, has not led to any predictive model able to recognize HBsAg seroclearance or seroconversion with sufficient accuracy to be usefully employed as an easy-to-use tool in the clinical setting. In the present study, the ANN was found to be superior to linear discriminant analysis as well as qHBsAg and HBV DNA levels both in the training group and non-inferior to linear discriminant analysis in the testing group, and very reliable in identifying HBsAg seroclearance. The better performance of ANN supported the postulation that HBsAg seroclearance was a complex, multidimensional nonlinear function [18], [19]. Our model was able to give a more precise estimate of HBsAg seroclearance on the basis of serum-based data routinely available in the clinical setting.

Our previous study showed low qHBsAg levels and increased rate of qHBsAg decline could predict HBsAg seroclearance [15]. By selecting these two clinical parameters and entering them into building the ANN and LRM, the accuracy of low qHBsAg in predicting HBsAg seroclearance was further increased (AUROC 0.847, 95%CI = 0.797–0.889). Accompanied with qHBsAg level decreasing gradually over times, lower levels of qHBsAg or rapid reduction rate of qHBsAg would eventually lead to HBsAg seroclearance or seroconversion [16]. Another important finding was the HBV DNA level and their reductions, which had previously been considered as powerful predictors for HBsAg seroclearance in pre-qHBsAg era [6], [8]. Liu et al. found that decrease in HBV DNA levels was the most important predictor of HBsAg seroclearance [8]. However, the predictability of HBsAg seroclearance increased greatly when they added the qHBsAg level into consideration [13]. In the present study, we compared the combination of the above predictors (ANN and LRM), as well as the separate predictors (qHBsAg and HBV DNA), respectively. Under these circumstances, use of the present ANN for HBsAg seroclearance, except for ANN for HBsAg seroconversion, could lead to an improvement in diagnostic accuracy and in tailoring the best individual clinical management.

The ANN for HBsAg seroconversion (AUROC 0.757) was inferior to that for HBsAg seroclearance. One of the potential reasons was the relatively small sample size (n = 63) which could affect the performance of ANN [22]. Nonetheless, given the rarity of HBsAg seroconversion, it would be difficult to recruit more subjects for a more thorough analysis. Another important reason was due to the lacking of significant predictors besides of the currently-available qHBsAg [24]. A good model for predicting HBsAg seroconversion remains to be discovered. Similarity, in genotype C subgroup, the performance of the ANN in predicting HBsAg seroclearance was not statistically significant and possibly underpowered since genotype C only comprised approximately one-third of the total patient cohort. A validation study concentrating on genotype C patients could be considered in the future.

Our study was limited by ANN being built and tested on a single center cohort, and it could thus be argued that data originating from other centers might lead to different conclusions. However, we believe that this should not be considered as a shortcoming since the distinctive characteristic of the ANN is that it can learn through examples making the prediction of HBsAg seroclearance, even in HBsAg seroconversion, feasible on datasets from other sources.

In conclusion, ANN could accurately predict spontaneous HBsAg seroclearance in HBeAg-negative CHB patients, on the basis of easily available serum data within a shorter period of no more than 3 years. ANN for HBsAg seroclearance was superior to the conventional statistical linear approach and it could be used in predicting the outcome of CHB. The performance of ANN for HBsAg seroclearance can be further improved by including new cases from other centers due to the unique ability of learning of neural networks.

## Supporting Information

Table S1 **Baseline characteristics of the study population stratified by HBsAg seroclearance subgroups.**
(DOC)

Table S2 **Baseline characteristics of the study population stratified by HBsAg seroconversion subgroups.**
(DOC)

Table S3 **Baseline characteristics of the study population, stratified by HBsAg seroclearance and seroconversion.**
(DOC)

Table S4 **Characteristics of the study population, stratified by HBsAg seroclearance or not.**
(DOC)

Table S5 **Characteristics of the study population, stratified by HBsAg seroconversion or not.**
(DOC)
