# Supplementary material for: Artificial Neural Network Accurately Predicts Hepatitis B Surface Antigen Seroclearance
Source: PLoS One. 2014 Jun 10;9(6):e99422. doi: 10.1371/journal.pone.0099422 (PMC4051672; doi:10.1371/journal.pone.0099422)
Supplement: Table S1 — Baseline characteristics of the study population stratified by HBsAg seroclearance subgroups. (DOC) [file pone.0099422.s001.doc]

| Table S1. Baseline characteristics of the study population stratified by HBsAg seroclearance subgroups. | | | | | |
| --- | --- | --- | --- | --- | --- |
| Variables | Training  (n = 284) | Testing in B genotype  (n = 141) | Testing in C genotype  (n = 53) | P value$ | P value£ |
| Age (years) | 48.4 ± 10.8 | 46.9 ± 11.9 | 47.1 ± 9.6 | 0.187 | 0.410 |
| Male gender (%) | 200 (70.4) | 111 (78.7) | 37 (69.8) | 0.069 | 0.929 |
| ALT (IU/L) | 28.0 ± 17.4 | 27.6 ± 15.4 | 28.3 ± 16.6 | 0.803 | 0.909 |
| Bilirubin (µmol/L) | 13.6 ± 9.2 | 14.1 ± 7.2 | 13.8 ± 10.6 | 0.586 | 0.898 |
| qHBsAg (log10 IU/ml)* | 1.94 ± 1.33 | 2.14 ± 1.22 | 1.94 ± 1.33 | 0.131 | 0.976 |
| HBV DNA (log10 IU/ml)* | 2.74 ± 1.21 | 3.08 ± 1.20 | 2.65 ± 1.47 | 0.008 | 0.631 |
| qHBsAg (log10 IU/ml)§ | 1.53 ± 1.43 | 1.79 ± 1.41 | 1.49 ± 1.48 | 0.097 | 0.833 |
| HBV DNA (log10 IU/ml)§ | 2.53 ± 1.28 | 2.90 ± 1.32 | 2.57 ± 1.44 | 0.007 | 0.818 |
| qHBsAg reduction (log10 IU/ml)¶ | 0.40 ± 0.52 | 0.34 ± 0.49 | 0.51 ± 0.57 | 0.276 | 0.182 |
| HBV DNA reduction (log10 IU/ml)¶ | 0.22 ± 0.94 | 0.23 ± 0.98 | 0.16 ± 0.97 | 0.872 | 0.709 |
| *Time point 3 years. §Time point 2 years. ¶Time point 3 to 2 years. Time point is defined as the period before HBsAg seroclearance: 0 year indicates date of seroclearance (baseline). $Between training group and testing in B genotype subgroup. £Between training group and testing in C genotype subgroup. | | | | | |
