# Supplementary material for: Artificial Neural Network Accurately Predicts Hepatitis B Surface Antigen Seroclearance
Source: PLoS One. 2014 Jun 10;9(6):e99422. doi: 10.1371/journal.pone.0099422 (PMC4051672; doi:10.1371/journal.pone.0099422)
Supplement: Table S2 — Baseline characteristics of the study population stratified by HBsAg seroconversion subgroups. (DOC) [file pone.0099422.s002.doc]

| Table S2. Baseline characteristics of the study population stratified by HBsAg seroconversion subgroups. | | | | | |
| --- | --- | --- | --- | --- | --- |
| Variables | Training  (n = 137) | Testing in B genotype  (n = 59) | Testing in C genotype  (n = 32) | P value$ | P value£ |
| Age (years) | 48.3 ± 10.5 | 46.2 ± 12.7 | 45.9 ± 8.9 | 0.227 | 0.236 |
| Male gender (%) | 97 (70.8) | 44 (74.6) | 26 (81.3) | 0.590 | 0.232 |
| ALT (IU/L) | 29.3 ± 19.2 | 27.5 ± 17.7 | 27.3 ± 17.6 | 0.531 | 0.595 |
| Bilirubin (µmol/L) | 12.6 ± 8.7 | 13.6 ± 7.6 | 14.3 ± 12.8 | 0.442 | 0.366 |
| qHBsAg (log10 IU/ml)* | 1.22 ± 1.13 | 1.45 ± 1.22 | 1.41 ± 0.89 | 0.208 | 0.388 |
| HBV DNA (log10 IU/ml)* | 2.23 ± 0.91 | 2.66 ± 1.13 | 2.00 ± 0.96 | 0.006 | 0.188 |
| qHBsAg (log10 IU/ml)§ | 0.57 ± 0.96 | 0.70 ± 1.19 | 0.73 ± 0.81 | 0.473 | 0.434 |
| HBV DNA (log10 IU/ml)§ | 1.79 ± 0.71 | 2.02 ± 0.89 | 1.83 ± 0.66 | 0.068 | 0.763 |
| qHBsAg reduction (log10 IU/ml)¶ | 0.62 ± 0.59 | 0.69 ± 0.55 | 0.70 ± 0.58 | 0.484 | 0.528 |
| HBV DNA reduction (log10 IU/ml)¶ | 0.43 ± 0.84 | 0.67 ± 1.05 | 0.24 ± 0.78 | 0.112 | 0.252 |
| *Time point 3 years. §Time point 2 years. ¶Time point 3 to 2 years. Time point is defined as the period before HBsAg seroclearance: 0 year indicates date of seroclearance (baseline). $Between training group and testing in B genotype subgroup. £Between training group and testing in C genotype subgroup. | | | | | |
