# Supplementary material for: Artificial Neural Network Accurately Predicts Hepatitis B Surface Antigen Seroclearance
Source: PLoS One. 2014 Jun 10;9(6):e99422. doi: 10.1371/journal.pone.0099422 (PMC4051672; doi:10.1371/journal.pone.0099422)
Supplement: Table S3 — Baseline characteristics of the study population, stratified by HBsAg seroclearance and seroconversion. (DOC) [file pone.0099422.s003.doc]

| Table S3. Baseline characteristics of the study population, stratified by HBsAg seroclearance and seroconversion. | | | | | | |
| --- | --- | --- | --- | --- | --- | --- |
| Variables | HBsAg seroclearance  (n = 203) | HBsAg non-seroclearance  (n = 203) | P value | HBsAg seroconversion  (n = 63) | HBsAg non-seroconversion  (n = 140) | P value |
| Age (years) | 48.7 ± 11.1 | 49.0 ± 10.7 | 0.791 | 46.1 ± 10.5 | 49.8 ± 11.2 | 0.027 |
| Male gender (%) | 143 (70.4) | 143 (70.4) | 0.999 | 45 (71.4) | 98 (70.0) | 0.837 |
| ALT (IU/L) | 28.6 ± 19.8 | 26.5 ± 13.0 | 0.208 | 31.3 ± 19.5 | 27.3 ± 19.9 | 0.186 |
| Bilirubin (µmol/L) | 13.7 ± 10.8 | 13.3 ± 6.2 | 0.706 | 12.7 ± 5.4 | 14.1 ± 12.4 | 0.403 |
| qHBsAg (log10 IU/ml)* | 1.26 ± 1.11 | 2.72 ± 1.10 | 0.001 | 1.59 ± 0.96 | 1.12 ± 1.15 | 0.006 |
| HBV DNA (log10 IU/ml)* | 2.25 ± 0.97 | 3.33 ± 1.32 | 0.001 | 2.49 ± 1.16 | 2.15 ± 0.86 | 0.022 |
| qHBsAg (log10 IU/ml)§ | 0.56 ± 0.93 | 2.60 ± 1.12 | 0.001 | 0.83 ± 0.90 | 0.45 ± 0.93 | 0.010 |
| HBV DNA (log10 IU/ml)§ | 1.83 ± 0.75 | 3.42 ± 1.35 | 0.001 | 1.92 ± 0.78 | 1.80 ± 0.74 | 0.325 |
| qHBsAg reduction (log10 IU/ml)¶ | 0.66 ± 0.60 | 0.16 ± 0.36 | 0.001 | 0.75 ± 0.56 | 0.62 ± 0.61 | 0.196 |
| HBV DNA reduction (log10 IU/ml)¶ | 0.40 ± 0.86 | -0.05 ± 1.01 | 0.001 | 0.60 ± 1.05 | 0.32 ± 0.75 | 0.040 |
| *Time point 3 years. §Time point 2 years. ¶Time point 3 to 2 years. Time point is defined as the period before HBsAg seroclearance: 0 year indicates date of seroclearance (baseline). | | | | | | |
