# Supplementary material for: Artificial Neural Network Accurately Predicts Hepatitis B Surface Antigen Seroclearance
Source: PLoS One. 2014 Jun 10;9(6):e99422. doi: 10.1371/journal.pone.0099422 (PMC4051672; doi:10.1371/journal.pone.0099422)
Supplement: Table S4 — Characteristics of the study population, stratified by HBsAg seroclearance or not. (DOC) [file pone.0099422.s004.doc]

| Table S4. Characteristics of the study population, stratified by HBsAg seroclearance or not. | | | | | | | | | | | | |
| --- | --- | --- | --- | --- | --- | --- | --- | --- | --- | --- | --- | --- |
| Variables | Training group (n = 284) | | | Testing group (n = 122) | | | Genotype B group (n = 141) | | | Genotype C group (n= 53) | | |
|  | HBsAg seroclearance  (n = 151) | HBsAg non-seroclearance  (n = 133) | P | HBsAg seroclearance  (n = 52) | HBsAg non-seroclearance  (n = 70) | P | HBsAg seroclearance  (n = 59) | HBsAg non-seroclearance  (n = 82) | P | HBsAg seroclearance  (n = 32) | HBsAg non-seroclearance  (n = 21) | P |
| Age (years) | 48.4 ± 11.2 | 48.5 ± 10.5 | 0.897 | 49.6 ± 11.0 | 49.8 ± 11.1 | 0.920 | 46.2 ± 12.7 | 47.4 ± 11.4 | 0.562 | 45.9 ± 8.9 | 48.9 ± 10.6 | 0.277 |
| Male gender (%) | 105 (69.5) | 95 (71.4) | 0.727 | 38 (73.1) | 48 (68.6) | 0.589 | 44 (74.6) | 67 (81.7) | 0.307 | 26 (81.3) | 11 (52.4) | 0.025 |
| ALT (IU/L) | 28.9 ± 20.3 | 27.0 ± 13.4 | 0.365 | 27.6 ± 18.6 | 25.4 ± 12.1 | 0.436 | 27.5 ± 17.7 | 27.7 ± 13.6 | 0.939 | 27.3 ± 17.6 | 29.8 ± 15.2 | 0.597 |
| Bilirubin (µmol/L) | 13.9 ± 11.3 | 13.3 ± 6.2 | 0.582 | 13.0 ± 9.2 | 13.4 ± 6.3 | 0.759 | 13.6 ± 7.6 | 14.5 ± 6.9 | 0.453 | 14.3 ± 12.8 | 13.1 ± 6.0 | 0.694 |
| qHBsAg (log10 IU/ml)* | 1.25 ± 1.13 | 2.71 ± 1.11 | 0.001 | 1.30 ± 1.09 | 2.74 ± 1.10 | 0.001 | 1.45 ± 1.22 | 2.63 ± 0.96 | 0.001 | 1.41 ± 0.89 | 2.76 ± 1.49 | 0.001 |
| HBV DNA (log10 IU/ml)* | 2.24 ± 0.92 | 3.32 ± 1.26 | 0.001 | 2.30 ± 1.12 | 3.36 ± 1.44 | 0.001 | 2.66 ± 1.13 | 3.38 ± 1.17 | 0.001 | 2.00 ± 0.96 | 3.66 ± 1.57 | 0.001 |
| qHBsAg (log10 IU/ml)§ | 0.60 ± 0.94 | 2.58 ± 1.11 | 0.001 | 0.47 ± 0.91 | 2.63 ± 1.15 | 0.001 | 0.70 ± 1.19 | 2.55 ± 0.99 | 0.001 | 0.73 ± 0.81 | 2.50 ± 1.57 | 0.001 |
| HBV DNA (log10 IU/ml)§ | 1.84 ± 0.75 | 3.31 ± 1.30 | 0.001 | 1.83 ± 0.75 | 3.62 ± 1.44 | 0.001 | 2.02 ± 0.89 | 3.51 ± 1.23 | 0.001 | 1.83 ± 0.66 | 3.60 ± 1.60 | 0.001 |
| qHBsAg reduction (log10 IU/ml)¶ | 0.40 ± 0.52 | 0.42 ± 0.62 | 0.001 | 0.80 ± 0.68 | 0.14 ± 0.39 | 0.001 | 0.69 ± 0.55 | 0.11 ± 0.27 | 0.001 | 0.70 ± 0.58 | 0.26 ± 0.46 | 0.006 |
| HBV DNA reduction (log10 IU/ml)¶ | 0.37 ± 0.83 | 0.04 ± 1.02 | 0.001 | 0.49 ± 0.94 | -0.22 ± 0.99 | 0.001 | 0.67 ± 1.05 | -0.07 ± 0.81 | 0.001 | 0.24 ± 0.78 | 0.06 ± 1.20 | 0.534 |
| *Time point 3 years. §Time point 2 years. ¶Time point 3 to 2 years. Time point is defined as the period before HBsAg seroclearance: 0 year indicates date of seroclearance (baseline). | | | | | | | | | | | | |
