# Supplementary material for: Artificial Neural Network Accurately Predicts Hepatitis B Surface Antigen Seroclearance
Source: PLoS One. 2014 Jun 10;9(6):e99422. doi: 10.1371/journal.pone.0099422 (PMC4051672; doi:10.1371/journal.pone.0099422)
Supplement: Table S5 — Characteristics of the study population, stratified by HBsAg seroconversion or not. (DOC) [file pone.0099422.s005.doc]

| Table S5. Characteristics of the study population, stratified by HBsAg seroconversion or not. | | | | | | | | | | | | |
| --- | --- | --- | --- | --- | --- | --- | --- | --- | --- | --- | --- | --- |
| Variables | Training group (n = 137) | | | Testing group (n = 66) | | | Genotype B group (n = 59) | | | Genotype C group (n= 32) | | |
|  | HBsAg seroconversion  (n = 43) | HBsAg non-seroconversion  (n = 94) | P | HBsAg seroconversion  (n = 20) | HBsAg non-seroconversion  (n = 46) | P | HBsAg seroconversion  (n = 27) | HBsAg non-seroconversion  (n = 32) | P | HBsAg seroconversion  (n = 14) | HBsAg non-seroconversion  (n = 18) | P |
| Age (years) | 46.1 ± 10.3 | 49.4 ± 10.5 | 0.093 | 46.1 ± 11.3 | 50.8 ± 12.5 | 0.160 | 44.6 ± 11.1 | 47.6 ± 13.9 | 0.359 | 44.0 ± 9.1 | 47.4 ± 8.8 | 0.289 |
| Male gender (%) | 28 (65.1) | 69 (73.4) | 0.322 | 17 (85.0) | 29 (63.0) | 0.074 | 22 (81.5) | 22 (68.8) | 0.263 | 9 (64.3) | 17 (94.4) | 0.030 |
| ALT (IU/L) | 33.6 ± 20.6 | 27.3 ± 18.3 | 0.075 | 26.4 ± 16.2 | 27.3 ± 23.1 | 0.868 | 31.2 ± 18.8 | 24.3 ± 16.5 | 0.144 | 31.1 ± 21.1 | 24.3 ± 14.2 | 0.285 |
| Bilirubin (µmol/L) | 12.5 ± 5.1 | 12.6 ± 10.0 | 0.975 | 13.2 ± 6.2 | 17.2 ± 16.0 | 0.280 | 13.5 ± 5.8 | 13.7 ± 8.9 | 0.931 | 11.6 ± 5.1 | 16.3 ± 16.4 | 0.313 |
| qHBsAg (log10 IU/ml)* | 1.63 ± 0.93 | 1.04 ± 1.16 | 0.004 | 1.50 ± 1.04 | 1.29 ± 1.12 | 0.474 | 1.82 ± 1.05 | 1.14 ± 1.28 | 0.032 | 1.43 ± 0.93 | 1.39 ± 0.89 | 0.909 |
| HBV DNA (log10 IU/ml)* | 2.52 ± 1.06 | 2.10 ± 0.80 | 0.012 | 2.42 ± 1.38 | 2.25 ± 0.97 | 0.566 | 2.93 ± 1.37 | 2.43 ± 0.82 | 0.090 | 1.84 ± 0.99 | 2.12 ± 0.94 | 0.436 |
| qHBsAg (log10 IU/ml)§ | 1.00 ± 0.94 | 0.38 ± 0.91 | 0.001 | 0.46 ± 0.71 | 0.58 ± 0.95 | 0.634 | 1.05 ± 1.10 | 0.39 ± 1.20 | 0.046 | 0.77 ± 0.81 | 0.70 ± 0.83 | 0.818 |
| HBV DNA (log10 IU/ml)§ | 1.91 ± 0.81 | 1.74 ± 0.66 | 0.216 | 1.93 ± 0.75 | 1.92 ± 0.86 | 0.954 | 2.04 ± 0.86 | 2.01 ± 0.92 | 0.898 | 1.91 ± 0.82 | 1.78 ± 0.55 | 0.628 |
| qHBsAg reduction (log10 IU/ml)¶ | 0.63 ± 0.45 | 0.61 ± 0.65 | 0.868 | 0.98 ± 0.70 | 0.63 ± 0.53 | 0.040 | 0.71 ± 0.55 | 0.67 ± 0.55 | 0.827 | 0.74 ± 0.38 | 0.68 ± 0.69 | 0.794 |
| HBV DNA reduction (log10 IU/ml)¶ | 0.60 ± 0.96 | 0.36 ± 0.77 | 0.140 | 0.61 ± 1.24 | 0.24 ± 0.72 | 0.156 | 0.97 ± 1.21 | 0.41 ± 0.83 | 0.053 | 0.03 ± 0.57 | 0.38 ± 0.89 | 0.238 |
| *Time point 3 years. §Time point 2 years. ¶Time point 3 to 2 years. Time point is defined as the period before HBsAg seroclearance: 0 year indicates date of seroclearance (baseline). | | | | | | | | | | | | |
